# A Novel RAYM_RS09735/RAYM_RS09740 Two-Component Signaling System Regulates Gene Expression and Virulence in *Riemerella anatipestifer*

**DOI:** 10.3389/fmicb.2017.00688

**Published:** 2017-04-21

**Authors:** Ying Wang, Ti Lu, Xuehuan Yin, Zutao Zhou, Shaowen Li, Mei Liu, Sishun Hu, Dingren Bi, Zili Li

**Affiliations:** ^1^Department of Preventive Veterinary Medicine, College of Veterinary Medicine, Huazhong Agricultural UniversityWuhan, China; ^2^State Key Laboratory of Agricultural Microbiology, Huazhong Agricultural UniversityWuhan, China

**Keywords:** *Riemerella anatipestifer*, *RAYM_RS09735/RAYM_RS09740*, two-component signaling system, virulence, RNAseq

## Abstract

The Gram-negative bacterium *Riemerella anatipestifer* is an important waterfowl pathogen, causing major economic losses to the duck-producing industry. However, little is known of the virulence factors that mediate pathogenesis during *R. anatipestifer* infection. In this study, RAYM_RS09735 and RAYM_RS09740 were predicted to form a two-component signaling system (TCS) through bioinformatics analysis. This TCS was highly conserved across the *Flavobacteriaceae*. A mutant YMΔRS09735/RS09740 strain was constructed to investigate the role of the RAYM_RS09735/RAYM_RS09740 TCS in *R. anatipestifer* virulence and gene regulation. The median lethal dose (LD_50_) of YMΔRS09735/RS09740 was found to be >10^11^ CFU, equivalent to that of avirulent bacterial strains. The bacterial abundances of the YMΔRS09735/RS09740 strain in the heart, brain, liver, blood, and spleen were significantly lower than that of the wild-type *R. anatipestifer* YM strain. Pathological analysis using hematoxylin and eosin staining showed that, compared to the wild-type, the mutant YMΔRS09735/RS09740 strain caused significantly less virulence in infected ducklings. RNAseq and real-time PCR analysis indicated that the RAYM_RS09735/RAYM_RS09740 TCS is a PhoP/PhoR system. This is a novel type of TCS for Gram-negative bacteria. The TCS was also found to be a global regulator of expression in *R. anatipestifer*, with 112 genes up-regulated and 693 genes down-regulated in the YMΔRS09735/RS09740 strain (~33% genes demonstrated differential expression). In summary, we have reported the first PhoP/PhoR TCS identified in a Gram-negative bacterium and demonstrated that it is involved in virulence and gene regulation in *R. anatipestifer*.

## Introduction

The disease riemerella anatipestifersis, caused by the Gram-negative bacterium *Riemerella anatipestifer*, occurs primarily in 1–8-week-old ducks but is most common in more susceptible 2–3-week-old ducklings. It is currently the most economically damaging bacterial infection affecting the global duck industry. Symptoms are characterized by fibrinous pericarditis, glissonitis, airbag inflammation, and meningitis (Segers et al., 1993). A total of 21 *R. anatipestifer* serotypes have been identified, with no significant cross-protection reported, making it difficult to control the disease through vaccination (Pathanasophon et al., 1995, 2002). *R. anatipestifer* serotypes 1, 2, and 10 are responsible for most major outbreaks in China. In recent years, many *R. anatipestifer* strains with differing virulence have been isolated from duck farms in China (Yuan et al., 2011; Wang et al., 2015; Zhang et al., 2015; Song et al., 2016). Additionally, several virulence factors have been identified that associate with disease severity, including VapD (Chang et al., 1998), CAMP cohemolysin (Crasta et al., 2002), outer membrane protein A (OmpA; Hu et al., 2011), Nicotinamidase PncA (Wang et al., 2016), and putative genes associated with lipopolysaccharide (LPS) synthesis (Yu et al., 2016).

Bacteria sense and adapt to their environment via two-component signaling systems (TCSs). A paired TCS typically has a sensing histidine kinase (HK) coupled to a response regulator (RR). The sensing of a signal by the HK leads to autophosphorylation on a histidine residue. Subsequent transfer of the phosphate to an aspartate residue on the cognate RR facilitates the binding of the RR to its specific DNA. Each phosphorylated RR regulates specific genes that enable individual bacteria to sense environmental factors and respond to stresses (Stock et al., 2003; Mike et al., 2014). TCSs are present in nearly all sequenced bacterial genomes, as well as in some fungal, archaeal, and plant species (Skerker et al., 2008).

TCSs are involved in the regulation of a variety of important biological functions in bacteria. In particular, PhoP/PhoQ TCSs control the transcription of key virulent genes in several bacterial pathogens, including *Escherichia coli, Shigella* spp. (Tobe, 2008), *Yersinia pestis* (O'Loughlin et al., 2010), and *Salmonella typhimurium* (Tran et al., 2016). TCSs also control the expression of *Pneumococcal* surface antigen A (PsaA), subsequently regulating virulence and resistance to oxidative stress in *Streptococcus pneumonia* (McCluskey et al., 2004). Related two-component system also regulate genes that are essential to virulence and complex lipid biosynthesis. For example, SenX3, and RegX3 form a TCS that is involved in *Mycobacterium tuberculosis* virulence (Ryndak et al., 2008). The TCS is expressed during phosphate starvation and is required for phosphate uptake and aerobic respiration (Parish et al., 2003; Haydel et al., 2012). Additionally, in TB, the TCS PrrAB is required during early intracellular infection (Haydel et al., 2012) and the TCS MprAB responds to envelope stress by regulating stress-responding and virulence-associated genes (He et al., 2006; Pang et al., 2007).

The bacterium *R. anatipestifer* YM was isolated in Yunmeng, Hubei province, China, and is a highly virulent strain classified as serotype 1 (Zhou et al., 2011). We have previously used *in vivo*-induced antigen technology (IVIAT) to identify *in vivo*-induced protein antigens from *R. anatipestifer*. This predicted the involvement of a putative TCS. To research the function of this TCS in *R. anatipestifer*, we constructed a mutant with the putative TCS genes, *RAYM_RS09735* and *RAYM_RS09740*, deleted to investigate their biological characteristics. We found that RAYM_RS09735/RAYM_RS09740 form a PhoP/PhoR TCS, the first reported such TCS in Gram-negative bacteria. We confirmed that the RAYM_RS09735/RAYM_RS09740 TCS is an important global transcription regulator and regulates the expression of virulence-associated genes in *R. anatipestifer*. This may provide the theoretical basis for further study into the molecular pathogenesis of *R. anatipestifer* and facilitate the design of genetically engineered vaccines against *R. anatipestifer*.

## Materials and methods

### Analysis of RAYM_RS09735 and RAYM_RS09740 homology in *R. anatipestifer*

The bacterial strains and plasmids used in this study are listed in Table 1. Primers used in this study are listed in Table 2. *In vivo*-induced antigen technology (IVIAT) was then used to characterize potential virulence factors that are expressed in ducks during infection with *R. anatipestifer*. A genomic DNA library of *R. anatipestifer* was screened, demonstrating *in vivo*-induced increased expression of genes with two ORFs, *RAYM_RS09735* and *RAYM_RS09740* (data not shown). RAYM_RS09735 and RAYM_RS09740 were predicted to be a histidine protein kinase (HK) and a response regulator (RR), respectively. To investigate *RAYM_RS09735* and *RAYM_RS09740* homology among different *R. anatipestifer* strains, the open reading frames of *RAYM_RS09735* and *RAYM_RS09740* were amplified from the wild-type *R. anatipestifer* YM strain. The PCR product was cloned into pMD-18T vectors and sequenced. Homologous amino acid sequences were identified by searching the GenBank database using BLASTX. The resulting alignments were used for the construction of a phylogenetic tree using neighbor-joining (NJ) and were further analyzed using MEGA v6.06 software.

**Table 1:** **Bacterial strains and plasmids used in this study**.

**Strains**	**Description**	**Source or references**
**STRAINS**		
*R. anatipestifer* YM	*R. anatipestifer* wild-type strain (serotype 1)	Lab collection (Zhou et al., 2011)
YMΔRS09735/RS09740	Mutant strain, derived from *R. anatipestifer* YM, in which the *RAYM_RS09735/RAYM_RS09740* genes were inactivated by Spec^R^ cassette insertion	This study
*E.coli* DH5α	F-φ80 lac ZΔM15 Δ(lacZYA-arg F) U169 endA1 recA1 hsdR17(rk-,mk+) supE44λ- thi -1 gyrA96 relA1 phoA	Lab collection
*E.coli* x7213	Thi-1 thr-1 leuB6 glnV44 fhuA21 lacY1 recA1 RP4-2-Tc::Mu λpir ΔasdA4 Δzhf-2::Tn10	Lab collection
**PLASMIDS**		
pMD-18T	Clone vector,Amp^R^	Life Technology
pIC333	Source of Spec^R^ cassette	Lab collection
pRE112	Suicide vector, sacB mobRP4 R6K ori Cm^R^	Life Technology
pRE112-LSR	Suicide vector with Spec^R^ and homology arms for homologous recombination	This tudy

**Table 2:** **Primers used in this study**.

**Name**	**Sequence (5′–3′)**
**PRIMERS FOR CONSTRUCTING SUICIDE PLASMID AND *RAYM_* RS09735/RAYM_RS09740 GENES MUTANT**	
RAYM_RS09735/RAYM_RS09740 L-F	CGGTACC TTTTAACTATTGTATTTATTTAC
RAYM_RS09735/RAYM_RS09740 L-R	CTTTTAAAACTACTGTCCAGTAGATTGATAA
Spec^R^ F	TATCAATCTACTGGACAGTAGTTTTAAAAG
Spec^R^ R	AAAACAACTTCAAAACAGTGGAACGAAAAC
RAYM_RS09735/RAYM_RS09740 R-F	GTTTTCGTTCCACTGTTTTGAAGTTGTTTT
RAYM_RS09735/RAYM_RS09740 R-R	TGAGCTCGGAAGGAAGCGTCTTGCAATATT
**PRIMERS FOR IDENTIFYING THE *RAYM_RS09735/RAYM_RS09740* GENES MUTANT**	
Check F	AAAGGTATGGGAATGGAG
Check R	GCCGTCTGTAGCAAGGGT
16S rRNA F	AGAGTTTGATCCTGGCTCAGGATC
16S rRNA R	ACGGCTACCTTGTTACGACTTAGC
**PRIMERS FOR qRT-PCR**	
RS09730 F	GAGCTTTGGGGTAGGGTAGA
RS09730 R	TTCCACCACCCAATTCCTGT
RS09735 F	GGTATGGGAATGGAGCCTCA
RS09735 R	GCCCCAAACCTTGACCTTTT
RS09740 F	ACGGCGAGGAAGGTCTTAAA
RS09740 R	CGTCTGCACCTAACTGATAACC
RS09745 F	AAATCCGCAACTTCGAGACG
RS09745 R	CCTCTGGCGTAGGAAAAGGA
RS05555 F	AGCTCTCTTGGATTGGCACT
RS05555 R	ATGCTCCCAAATACTCGGCT
RS04585 F	AAACGACGCTCCAATGCTTT
RS04585 R	GGAGGTGGAGAAGGAGCAAT
RS07825 F	AGTTGTAGGCGACCGAGAAA
RS07825 R	GGGAGGAATAGCTTTTCGGG
RS03930 F	AATGAGTGTCTTCAGGGGCA
RS03930 R	ACAGTCTGACGCTGCCTATT
RS04770 F	GGTTCCGAGAATTGGTTGCT
RS04770 R	TAGCCCTCAGTTTTGGAGCA
RS08905 F	GTCGTCCCCTCCTAAGTGTG
RS08905 R	GACTGTGGTGGTGGTACTCA
RS02680 F	GCCTCTACCTTCAGTCCAGA
RS02680 R	AGCGAGTTATTCCTGGACCT
RS08330 F	TCCCACATTGTAGGCAGGAG
RS08330 R	TGGAACACCTGGGCAGATAG
RS03960 F	AATCCAAGAGACAACCGCAC
RS03960 R	ACTCGTCCGTTAGCACTTCT
RS04760 F	AGCGATGAGAAAAGCAGCTC
RS04760 R	TCTCGACTAGGACAATGCCG
pstS F	AGTGCTACCAGTGATGGATGA
pstS R	ATCCATTCCCAACCCCGAAA
hydrolase Nlp/P60 F	GCGTTGTAAGCGGCTTTACT
hydrolase Nlp/P60 R	ACTCACTGCCGCTCATAAGA
BLP F	CTGCAACATAGTTAGCTTCGG
BLP R	ACAGCTACTCCCTCTAACGC
DnaB F	AAACCCGCCCTAATAAACGC
DnaB R	GCTGGTTTGTGCTCCTTCTT

### Bacterial strains and animals

*R. anatipestifer* RA-YM was grown in trypticase soy broth (TSB) or on agar plates (Difco Laboratories, USA) at 37°C with 5% CO_2_. *Escherichia coli X7213* was cultured in Luria Bertani (LB) broth containing 50 μg/mL diaminopimelic acid (DAP), with shaking at 37°C overnight (Roland et al., 1999). When needed, antibiotics were used at the following concentrations: 50 μg/mL spectinomycin (Spec), 25 μg/mL chloramphenicol (Cm), and 100 μg/mL ampicillin (Amp). A portion of the bacterial colonies grown in LB medium were stored at −80°C with 15% glycerol.

### Plasmid construction

The suicide vector used to create the *R. anatipestifer* YM *RAYM_RS09735/RAYM_RS09740* mutant was based on pRE112. Briefly, sequences 800 bp upstream and 800 bp downstream of *RAYM_RS09735* and *RAYM_RS09740* were amplified by PCR using the primer pairs RAYM_RS09735/RAYM_RS09740L F/R and RAYM_RS09735/RAYM_RS09740R F/R. These contained *Kpn*I and *Sac*I restriction sites. A spectinomycin resistance (Spec^R^) cassette (1,185 bp) was PCR amplified from plasmid pIC333 using the primers Spec^*R*^ F and Spec^R^ R. These three fragments were then purified from an agarose gel and used as a PCR template at a 1:1:2 M ratio to join overlapping PCR products with the primers RAYM_RS09735L-F and RAYM_RS09740R-R. The final product was digested with *Kpn*I and *Sac*I enzymes and ligated into the pRE112 plasmid (Edwards et al., 1998) to yield suicide plasmid pRE112-LSR. This plasmid was used to delete *RAYM_RS09735* and *RAYM_RS09740*.

### Construction of the *R. anatipestifer* RAYM_RS09735/RAYM_RS09740 mutant

The *R. anatipestifer* YM RAYM_RS09735/RAYM_RS09740 mutant, termed YMΔRS09735/RS09740, was constructed via allelic exchange using the previously constructed suicide plasmid pRE112-LSR. The donor strain *X7213* was transformed with pRE112-LSR and grown overnight in LB medium supplemented with 50 μg/mL DAP, 50 μg/mL Spec, and 25 μg/mL Cm. The recipient *R. anatipestifer* YM strain was cultured in TSB medium to an OD_600_ of 0.4–0.5. One milliliter of the donor strain and 3 mL of receptor strain were centrifuged at 3,000 rpm/min for 5 min, and the pellet re-suspended in 1 mL TSB. Mixed cultures were then incubated on a TSA plate supplemented with 50 μg/mL DAP at 37°C for 24 h, facilitating the conjugation of the donor and receptor strains. Cultures were next streaked onto a TSA plate containing 50 μg/mL spectinomycin (Spec) to isolate the putative *R. anatipestifer* YM conjugants from the mixed strains. Single colonies were re-purified on TSA plates supplemented with 50 μg/mL Spec. The *R. anatipestifer RAYM_RS09735/RAYM_RS09740* mutant strain was screened and validated by PCR.

### Characteristics of the YMΔRS09735/RS09740 strain

The wild-type strain *R. anatipestifer* or the mutant strain YMΔRS09735/RS09740 was grown in TSB at 37°C for 12 h with shaking, respectively. Equal amounts of YM culture were transferred into fresh TSB (without serum) at a ratio of 1:100 (vol/vol) and incubated at 37°C with shaking at 200 rpm. Bacterial growth was measured as described previously (Hu et al., 2011) by counting the number of bacterial CFUs at 2 h intervals for 14 h.

### Determination of bacterial virulence and survival *in vivo*

To determine whether deletion of *RAYM_RS09735* and *RAYM_RS09740* influenced *R. anatipestifer* virulence, the median bacterial lethal dose (LD_50_) of the mutant strain YMΔRS09735/RS09740 was determined (Hu et al., 2010). Healthy Cherry Valley ducks were purchased from Chunjiang Duck Company (China) and hosted in an isolated animal room. All animal experiments and procedures were approved by the Research Ethics Committee, Huazhong Agricultural University, Hubei, China. A total of 75 ducks were divided randomly into 15 groups (five ducks per group). The respective bacterial strains were then injected into duck flippers at a dose of 10^5^–10^11^ CFU. Ducks in groups 1–7 were injected with 10^5^–10^11^ CFU of YM bacteria, and ducks in groups 8–14 were injected with 10^5^–10^11^ CFU of YMΔRS09735/RS09740 strain (Zou et al., 2015). The control group was injected with an equivalent volume of PBS.

For pathological histological examination, ducks from each group were sacrificed 48 h after injection with 5 × 10^6^ CFU of each bacterial strain diluted in 0.5 mL PBS. Heart, liver, brain, and spleen samples were collected. Tissues were then fixed in formalin, sectioned, and stained with hematoxylin and eosin.

Twelve 12-day-old ducks were randomly divided into three groups (four ducks per group) and infected with either 5 × 10^6^ CFU of the wild-type *R. anatipestifer* strain or the mutant strain YMΔRS09735/RS09740. Blood, heart, liver, brain, and spleen samples were collected 48 h after injection. Tissues were homogenized in 5 mL PBS, serially diluted, and plated on TSA plates to assess the number of viable bacteria.

### Extraction of total RNA from *R. anatipestifer in vitro*

The mutant strain YMΔRS09735/RS09740 or wild-type strain YM was grown in TSB to log phase, respectively, and then harvested in no more than 3 mL culture by centrifugation at 4000–5000 × g for 5–10 min at 4°C. Total RNA was extracted using a Bacterial RNA Kit (Omega). A NANODROP 2000c (Nanodrop) was used to measure the concentration and quality of bacterial RNA.

### RNAseq library construction

A total of 1 μg RNA per sample was used for RNA sample preparations. Sequencing libraries were generated using a NEBNext® Ultra™ RNA Library Prep Kit for Illumina® (NEB), following the manufacturer's recommendations. Briefly, mRNA was purified from total RNA using poly-T oligo-attached magnetic beads. Fragmentation was carried out using divalent cations under elevated temperature in NEBNext First Strand Synthesis Reaction Buffer (5X). The first strand of cDNA was synthesized using a random hexamer primer and M-MuLV Reverse Transcriptase (RNase H–). Second strand cDNA synthesis was then subsequently performed using DNA Polymerase I and RNase H. Any remaining overhangs were converted into blunt ends via exonuclease/polymerase. After adenylation of the 3′ ends of DNA fragments, adaptors with a hairpin loop structure were ligated in preparation for hybridization. To select cDNA fragments by length, library fragments were purified with a AMPure XP system (Beckman Coulter). Next, 3 μL USER Enzyme (NEB) was incubated with size-selected, adaptor-ligated cDNA at 37°C for 15 min followed by 5 min at 95°C before PCR. PCR was performed using a Phusion High-Fidelity DNA polymerase, a universal PCR primer set and an Index (X) primer. Finally, PCR products were purified and the library quality assessed using an Agilent Bioanalyzer 2100 system (Agilent).

### RNAseq differential expression analysis

HTSeq v0.6.1 was used to count the number of reads that mapped to each gene and then gene expression was calculated using an RPKM method (Reads Per kb per Million reads). Differential expression analysis was performed using DESeq. A q-value (or FDR) <0.001& and a log_2_ fold-change >1 were set as the thresholds for determining significantly differential expression.

### Go enrichment analysis

Gene Ontology (GO) enrichment analysis of differentially expressed genes was performed using the Bioconductor package GOseq, with a gene length bias correction. GO functional analysis provided GO functional classification annotation for DEGs, as well as GO functional enrichment analysis. The Gene Ontology database used can be found at http://www.geneontology.org/.

### KEGG pathway enrichment analysis

As different genes cooperate with each other to exercise biological functions, pathway-based analysis can help further understand the biological functions of genes. We used KOBAS to test for the statistical enrichment of differential expression genes in the KEGG pathway data set (http://www.genome.jp/kegg/).

### Quantitative real-time PCR analysis

For quantitative real-time PCR (qRT-PCR) validation experiments, 10 genes were randomly selected to assess the RNAseq data (Table 2). For this analysis, 1 μg of RNA was reverse-transcribed to cDNA using the PrimeScript™ RT regent kit with gDNA Eraser (Takara), according to manufacturer's instructions. cDNA was diluted 10-fold and used for real-time PCR analysis using a Bio-Rad CFX96™ System and signal detection protocols in accordance with the manufacturer's instructions (TaKaRa). *DnaB* was used as an endogenous control. Primers used for the qRT-PCR are described in Table 2. Data analysis was performed using GraphPad Prism v 5.0 Software (GraphPad).

### Statistical analysis and data records

Student's *t*-tests were used to compare gene expression data. *P*-values of ≤0.05 were considered significant. RNAseq original data were uploaded to the NCBI Short Read Archive (SRA) with study accession number SRP096616 (http://www.ncbi.nlm.nih.gov/sra).

## Results

### Identification of the RAYM_RS09735 and RAYM_RS09740 genes in *R. anatipestifer*

**Figure 1:**
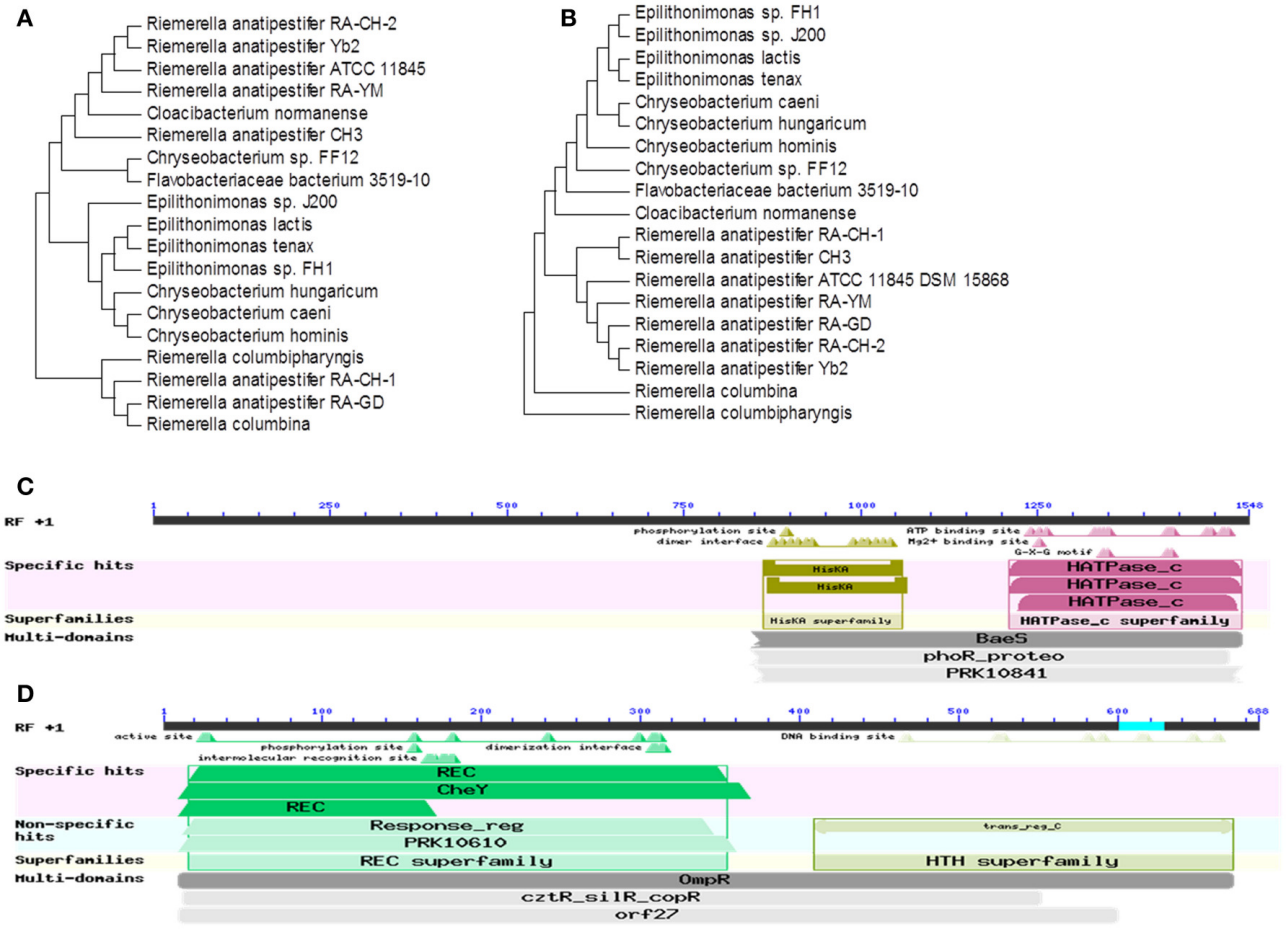
**Analys is of the RAYM_RS09735 and RAYM_RS09740 homology in ***R. anatipestifer*****. The bootstrap percentage from 1,000 replicates is indicated at each node. Rooted neighbor-joining tree of the amino acid sequences of RAYM_RS09735 **(A)** and RAYM_RS09740 **(B)**. Bioinformatics analysis the conserved domains of RAYM_RS09735 **(C)** and RAYM_RS09740 **(D)**.

Homologous amino acid sequences were identified by searching the GenBank database using BLASTX. A phylogenetic tree was constructed containing 19 amino homologous acid sequences (Figures 1A,B). RAYM_RS09735 and RAYM_RS09740 share more than 98% sequence identity with seven other *R. anatipestifer* strains. In addition, RAYM_RS09735 and RAYM_RS09740 shared 70% identity with species of *Flavobacteriaceae*, including *Cloacibacterium, Epilithonimonas*, and *Chryseobacterium*. Our results demonstrated that RAYM_RS09735 and RAYM_RS09740 are not only highly conserved in *R. anatipestifer*, but also conserved across the *Flavobacteriaceae* in general. Functional assessment predicted RAYM_RS09735 and RAYM_RS09740 to be elements of a two-component signaling system (TCS). TCS are typically composed of a sensor with histidine kinase activity and a cytoplasmic transcriptional regulator. RAYM_RS09735 was identified as a BaeS family histidine kinase, while RAYM_RS09740 was predicted to be an OmpR family transcriptional regulator (Figures 1C,D). Both members of the TCS had the same promoter. Additionally, RAYM_RS09735 was predicted to be a phosphate regulon sensor protein (PhoR).

### Characterization of a mutant YMΔRS09735/RS09740 strain

**Figure 2:**
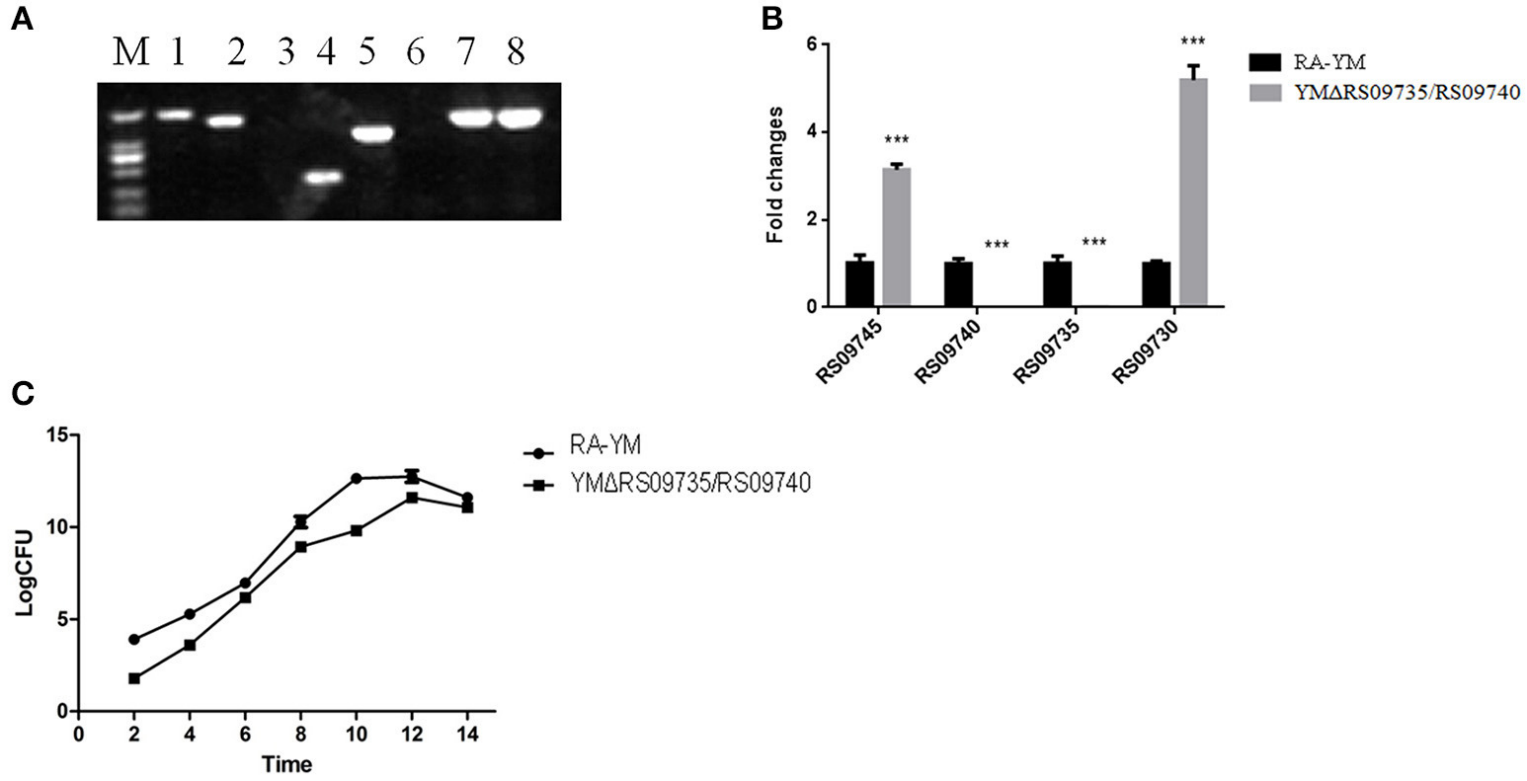
**Characteristics of YMΔRS09735/RS09740 strain. (A)** PCR amplification. M, DL2000; Lane 1, *RAYM_RS09735/RAYM_RS09740* was amplified from YMΔRS09735/RS09740 strain; Lane 2, *RAYM_RS09735/RAYM_RS09740* was amplified from RA-YM strain; Lane 3, delta genes were amplified from YMΔRS09735/RS09740 strain; Lane 4, delta genes were amplified from RA-YM strain; Lane 5, Spec^R^ cassette was amplified from YMΔRS09735/RS09740 strain; Lane 6, Spec^R^ cassette was amplified from RA-YM strain; Lane 7, 16S rRNA fragment was amplified from YMΔRS09735/RS09740 strain; Lane 8, 16S rRNA fragment was amplified from RA-YM strain. **(B)** Real-time PCR analysis. The flanks mRNA levels of *RAYM_RS09735/RAYM_RS09740* genes were measured. The changes of transcription were expressed as fold expression; **(C)** Bacterial growth curves. The mutant strain YMΔRS09735/RS09740 or wild-type strain RA-YM was grown on TSB medium, and growth of each strain was monitored by measuring the CFU/ml. ^***^P < 0.001.

The *RS09735/RS09740* gene was deleted from the chromosome of an *R. anatipestifer* YM strain via allelic exchange. The gene was replaced with a Spec^R^ cassette, allowing successful transfects to be drug selected. The mutant strain was further validated by PCR amplification of *RAYM_RS09735, RAYM_RS09740*, and 16S rRNA fragments from transconjugants (Figure 2A). Real-time PCR analysis confirmed that *RAYM_RS09735* and *RAYM_RS09740* transcription was completely abolished in the mutant strain. However, inactivation of the *RAYM_RS09735* and *RAYM_RS09740* genes led to significantly increased transcription of the chromosomally upstream gene *RAYM_RS09730* and the downstream gene *RAYM_RS09745* (Figure 2B). The *RAYM_RS09735* and *RAYM_RS09740* deletion mutant strain was designated YMΔRS09735/RS09740. Growth curve measurements revealed that growth in TSB was similar between the wild-type YM and mutant strains (Figure 2C). Transmission electron microscopy also showed that there were no significant changes in bacterial morphology in the RA-YM *RAYM_RS09735/RAYM_RS09740* mutant strain (data not shown).

### Pathogenicity of the YMΔRS09735/RS09740 mutant

**Figure 3:**
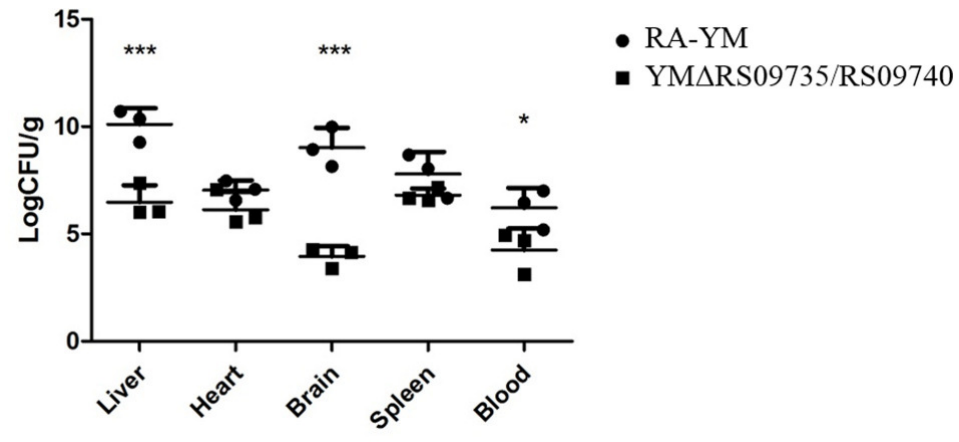
**Bacterial loadings in ***R. anatipestifer*** YM and YMΔRS09735/RS09740 in infected duckling tissues**. Twelve 12-day-old ducks infected with the wild-type *R. anatipestifer* strain or mutant strain YMΔRS09735/RS09740. Blood, heart, liver, brain, and spleen samples were collected at 48 h after injected flippers. ^*^P < 0.05, ^***^P < 0.001.

**Figure 4:**
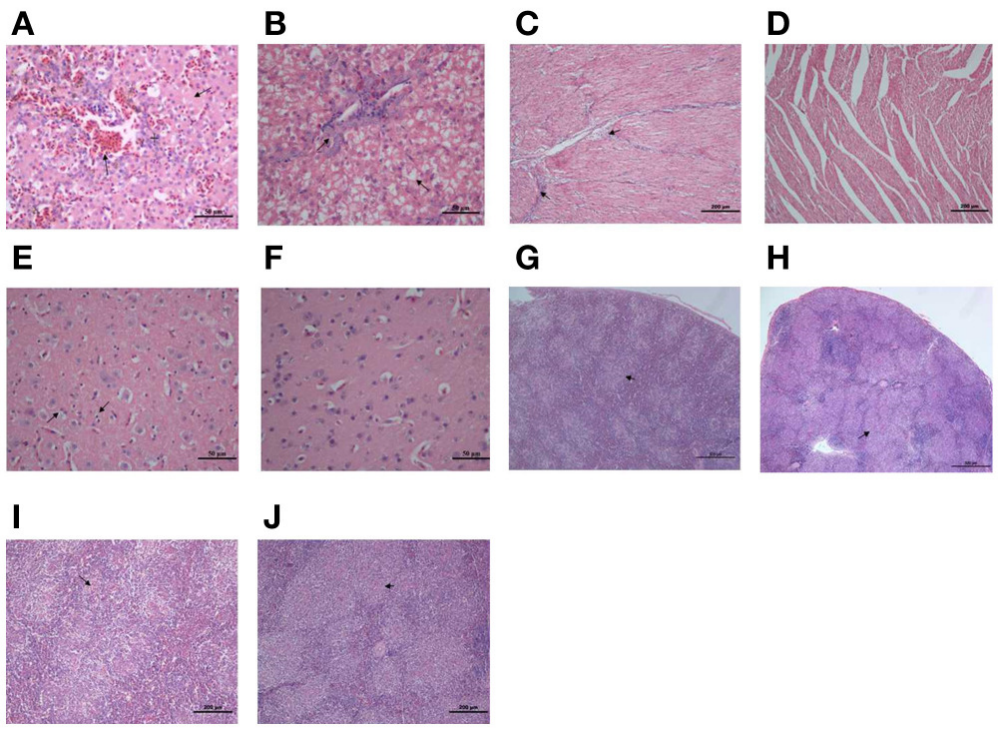
**Histopathological analysis of the duck tissues**. Liver, heart, brain, and spleen samples were collected from the infected ducks with *R. anatipestifer* YM strain **(A,C,E,G,I)** or YMΔRS09735/RS09740 strain **(B,D,F,H,J)** in 48 h post-challenge, fixed in 10% formalin, and subjected to hematoxylin and eosin staining for a histopathological examination. The “arrow” represents the obvious pathologic change in tissues.

Bacterial virulence was evaluated by median lethal doses (LD_50_) in 12-day-old Cherry Valley ducks. The LD_50_ for the mutant strain was greater than 10^11^ CFU, which was more than a 10^3^-fold attenuation in virulence compared to the wild-type YM strain (4 × 10^7^ CFU). These LD_50_ values demonstrate that the strain is almost avirulent in ducklings (Table 3). To further investigate the role of RAYM_RS09735 and RAYM_RS09740 in *in vivo* systemic infections, the bacterial loads in the liver, spleen, heart, brain, and blood from infected ducks were quantified. This showed that, in these organs, bacterial capacity was significantly decreased compared to ducks infected with the wild-type strain (Figure 3). Pathological histological analysis was also performed 48 h post-infection comparing the wild-type YM and YMΔRS09735/RS09740 strains. Hematoxylin and eosin staining revealed that the capacity of the YMΔRS09735/RS09740 mutant to damage the heart, brain, and spleen was significantly decreased relative to the wild-type strain, although mild liver damage was still observed (Figure 4). These results show that RAYM_RS09735 and RAYM_RS09740 significantly affect the virulence of *R. anatipestifer*.

**Table 3:** **Virulence of the wild-type ***R. anatipestifer*** YM and YMΔRS09735/RS09740 strain**.

**Strains**	**Challenge dose (CFU)**	**Survival/total**	**LD50 (CFU)**
*R.anatipestifer*	10^5^	5/5	4 × 10^7^
	10^6^	4/5	
	10^7^	3/5	
	10^8^	0/5	
	10^9^	0/5	
	10^10^	0/5	
	10^11^	0/5	
YMYMΔRS09735/RS09740	10^5^	5/5	>10^11^
	10^6^	5/5	
	10^7^	5/5	
	10^8^	5/5	
	10^9^	5/5	
	10^10^	5/5	
	10^11^	5/5	

### Function and GO enrichment analysis of the differentially expressed genes

**Figure 5:**
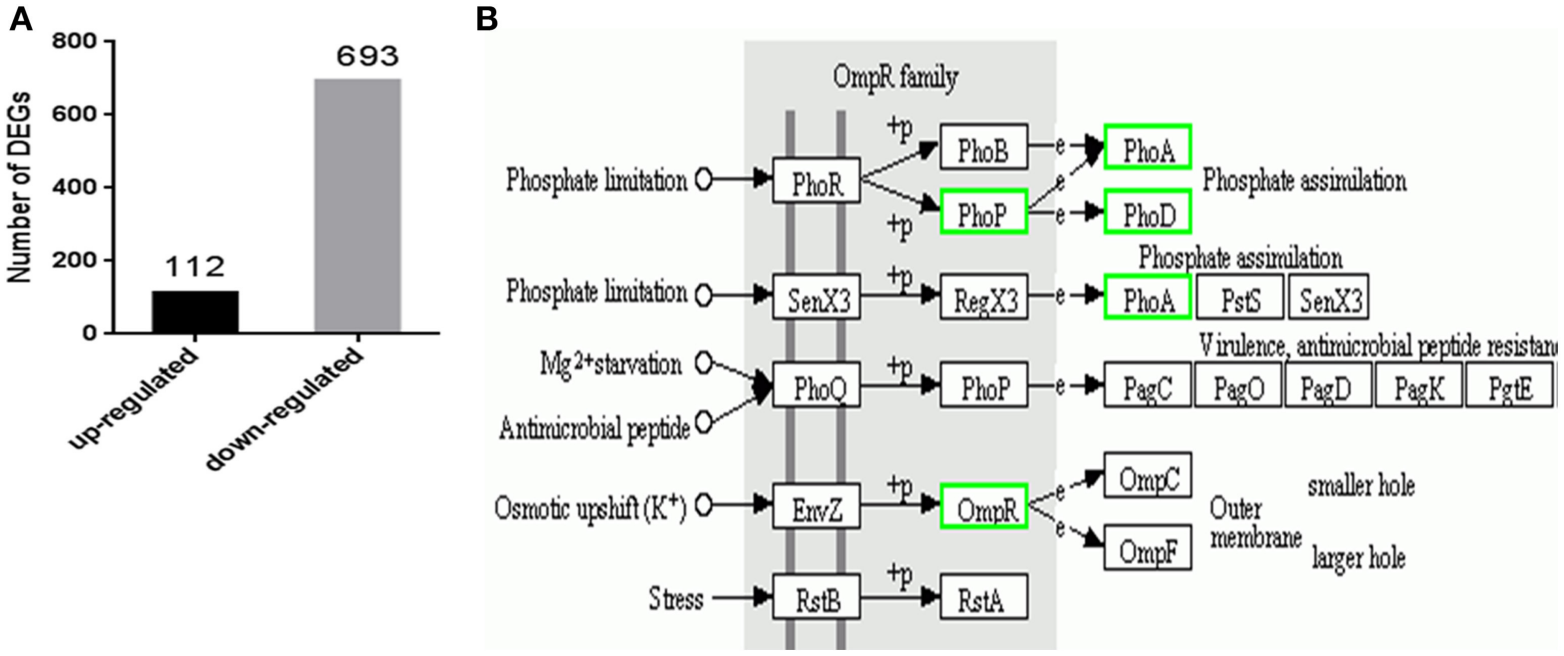
**Differential expression genes and functional classification of YMΔRS09735/RS09740 mutant strain. (A)** RNASeq analysis was performed to identify differentially expressed genes in mutant strain YMΔRS09735/RS09740. **(B)** RAYM_RS09740 in KEGG pathway from RNASeq analysis.

**Figure 6:**
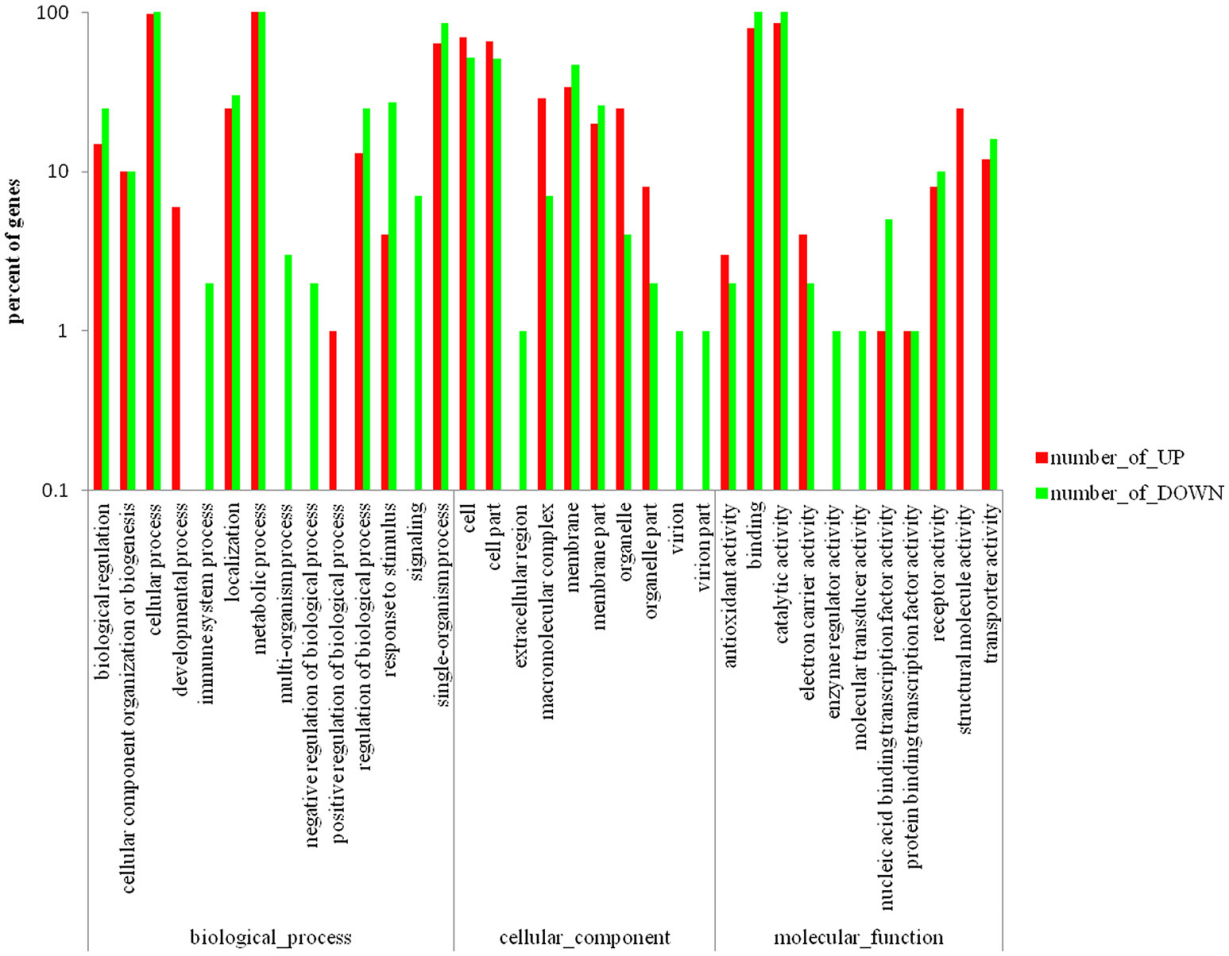
**Gene ontology enrichment analysis of the differentially expressed genes scattered to biological-process, cellular-component, and molecular-function, which including 21 pathways**.

**Figure 7:**
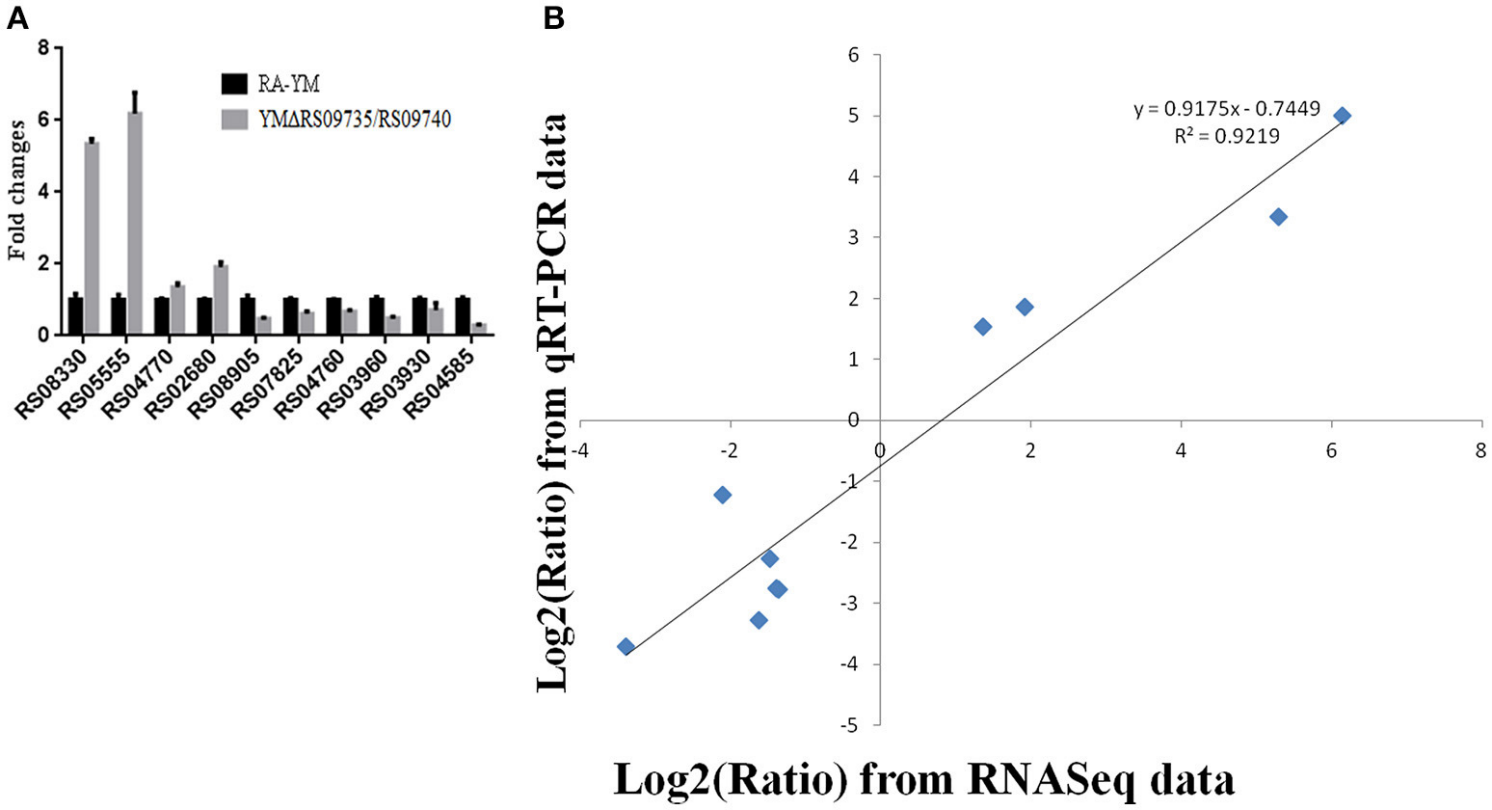
**Real-time PCR verification of differentially expressed genes in YMΔRS09735/RS09740 mutant strain. (A)** The fold expression changes of randomly selected 10 genes to validity of the RNAseq results. **(B)** Coefficient analysis of fold changes data between qRT-PCR and RNAseq. Ten different expression genes were selected from qRT-PCR.

Differentially expressed genes (DEGs) between the mutant YMΔRS09735/RS09740 and wild-type strains were identified using RNAseq. In total, 805 genes were found to be differentially expressed, with 112 genes upregulated (13.9%), and 693 genes downregulated (86.1%) in the mutant YMΔRS09735/RS09740 strain compared to the wild-type strain (Figure 5A). Of the ~2,000 genes identified in the *R. anatipestifer* genome, more than one third were differentially expressed. Further analysis of the RNAseq data using KEGG pathways predicted that RAYM_RS09735 and RAYM_RS09740 are components of a PhoP/PhoR TCS (Figure 5B). Gene Ontology (GO) enrichment analysis showed that differentially expressed genes can be found in a wide-variety of biological processes, cellular components, and molecular functions, including 21 distinct pathways (Figure 6). RNAseq was validated using qRT-PCR analysis of randomly selected 10 genes. Overall, the changes in expression of these randomly genes tested by qRT-PCR agreed with the direction determined by RNAseq (Figure 7A). Linear regression analysis was performed examining the fold-changes of the gene expression ratios between RNAseq and qRT-PCR, showing a significant positive correlation (Figure 7B).

We found that 11 genes had more than 4-fold higher expression in the mutant strain compared to the wild-type, eight of which encode hypothetical proteins. The three other genes encoded a carbohydrate-binding protein, a glycan metabolism protein (RagB), and a phosphate subunit transfer protein (PstS), respectively. We also found that several transcription factors, components of the CRISPR system, and additional putative proteins were also upregulated, with most upregulated genes involved in bacterial metabolism. In addition, 20 genes were found to be downregulated more than 4-fold. Of these, 13 genes encoded hypothetical proteins, while the other seven encoded RAYM_RS09735, RAYM_RS09740, a lipoprotein, a peptidoglycan hydrate (Nlp/P60), a DNA-binding protein, von Willebrand factor A, ATPase AAA, and an uncharacterized conserved protein. Other downregulated genes included several hypothetical proteins, transcription factors, and metabolic genes, in addition to multiple molecular chaperones and TonB-dependent receptors.

**Figure 8:**
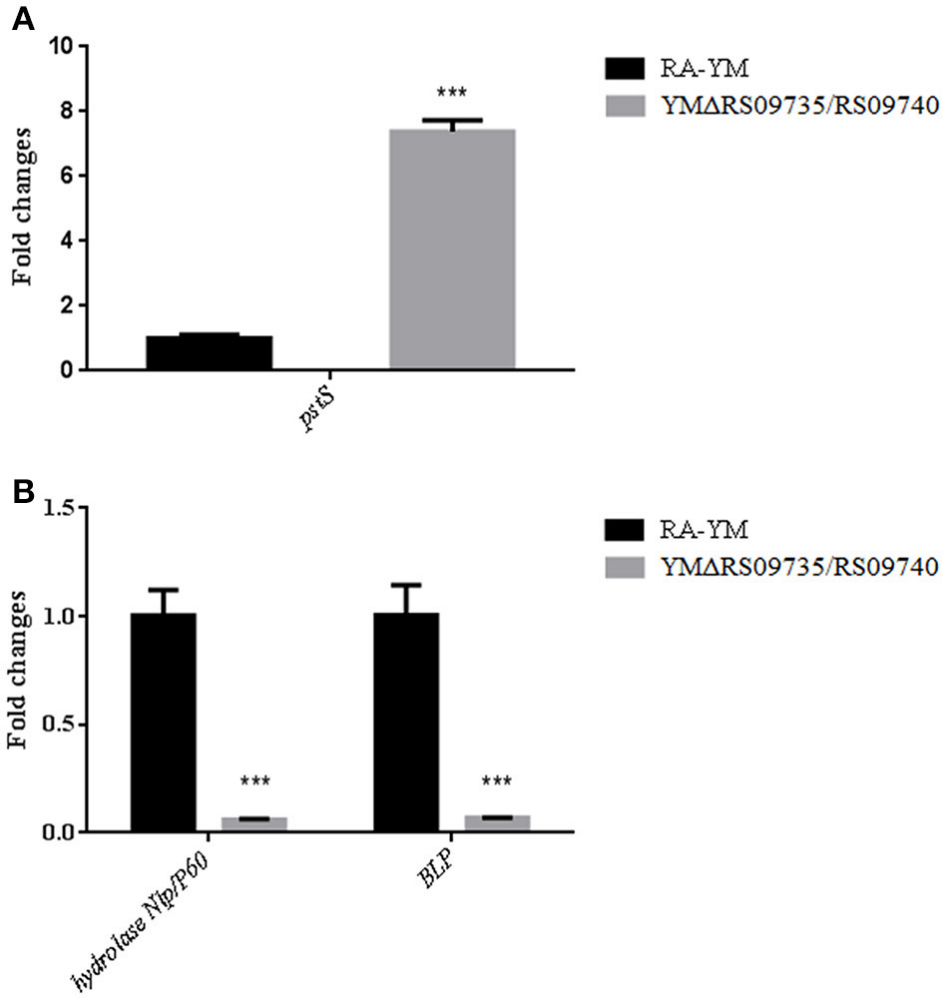
**Real-time PCR verification of three predicted virulence-associated genes expression in YMΔRS09735/RS09740 mutant strain. (A)** The fold expression changes of *pstS* gene in mutant strain. **(B)** The fold expression changes of two predicted virulent genes *hydrolase Nlp/P60* and *BLP* in mutant strain. ^***^P < 0.001.

Finally, we performed real-time PCR analysis of three predicted virulence-associated genes expression in the YMΔRS09735/RS09740 mutant strain (*pstS, BLP*, and *Nlp/P60*). We found that the mRNA expression of *pstS* was 8-fold higher (Figure 8A), consistent with our RNAseq data. *Bacterial lipoprotein* (*BLP*) and *hydrolase Nlp/P60* were found to be significantly downregulated (Figure 8B).

## Discussion

Bacterial two-component regulatory systems (TCSs), consisting of a sensing histidine kinase and a response regulator, mediate gene expression in response to environmental stimuli (Stock et al., 2003). To achieve this, histidine kinases sense environmental signals and autophosphorylate. This additional phosphate is then transferred to an aspartic acid residue on the corresponding response regulator (Hoch, 2000; Alm et al., 2006). The phosphorylated response regulator elicits a diverse range of downstream responses, including an enhancement of its DNA binding ability. This allows the TCS to modulate target gene expression (West and Stock, 2001; Lin et al., 2015). Using an *in vivo* antigen technology (IVIAT) to examine ducks infected with *R. anatipestifer*, we have identified a putative TCS involving the genes *RAYM_RS09740* and *RAYM_RS09735*. Bioinformatics analysis of these two genes, and their proteins, revealed that *RAYM_RS09740* and *RAYM_RS09735* encode a histidine kinase and response regulator, respectively. We also found that, like most TCSs, *RAYM_RS09735* and *RAYM_RS09740* have the same promoter and co-transcribe as an operon (Aggarwal et al., 2016). RAYM_RS09735 and RAYM_RS09740 possess all of the characteristic TCS domains essential to their biochemical activities and responses. RAYM_RS09735 and RAYM_RS09740 were predicted to belong to the OmpR and BaeS families, respectively, and RAYM_RS09735 was further predicted to be a phosphate regulon sensor protein (PhoR).

Our study aimed to investigate the RAYM_RS09735/RAYM_RS09740 TCS and explore its potential functions in *R. anatipestifer*. To better evaluate the role of RAYM_RS09735 and RAYM_RS09740 in *R. anatipestifer*, we constructed the mutant strain YMΔRS09735/RS09740 in which the *RAYM_RS09735* and *RAYM_RS09740* genes were mutated by deleting a 1400-bp fragment from the wild-type RA-YM strain. RT-PCR and qRT-PCR were used to validate the mutant strain (Figures 2A,B). We found that upstream and downstream genes were significantly down-regulated in the YMΔRS09735/RS09740 mutant, although the strain showed the same growth characteristics as the wild-type in TSB (Figure 2C).

LD_50_ analysis of the two strains demonstrated the *RAYM_RS09735/RAYM_RS09740* mutant had significantly reduced virulence. Indeed, the strain was almost avirulent to ducklings (Table 3). A normal *R. anatipestifer* infection is characterized by septicemia and the ability to colonize and develop in the tissues of their host. We found that the bacterial loads in the liver, spleen, heart, brain, and blood of ducks infected with the mutant YMΔRS09735/RS09740 strain were significantly lower than that of wild-type RA-YM infected ducklings (Figure 3). In addition, pathological histological analysis using hematoxylin and eosin staining showed that the mutant strain also induced significantly less damage to the heart, brain, and spleen, compared to the wild-type strain. There was still slight damage to the liver (Figure 4). Our results indicate that the *RAYM_RS09735* and *RAYM_RS09740* genes are important mediators of *R. anatipestifer* virulence during infection in ducklings. Therefore, RAYM_RS09740 was determined to be a response regulator, with a combined promoter affecting downstream genes. The protein also altered the expression of target genes. Our study therefore indicated that virulence factors in *R. anatipestifer* could be regulated by RAYM_RS09740.

RAYM_RS09735 and RAYM_RS09740 share up to 70% sequence identity with *Flavobacteriaceae* species, such as *Cloacibacterium, Epilithonimonas*, and *Chryseobacterium*. These species have been previously reported to exhibit drug resistance. Molecular docking analysis identified inhibitors of various RRs in OmpR family to affect this observed drug resistance. Therefore targeting TCSs has become an attractive option for the development of new drugs, particularly to combat *M. tuberculosis* (Banerjee et al., 2016). These studies also demonstrate that TCSs can be used as the theoretical basis for the study of drug resistance mechanisms in *Flavobacteriaceae*.

To investigate how RAYM_RS09735 and RAYM_RS09740 regulate gene expression, RNAseq analysis was performed to identify differentially expressed genes in the mutant YMΔRS09735/RS09740 strain. This revealed 112 genes that were upregulated (13.9%) and 693 genes that were down-regulated (86.1%) relative to the wild-type strain (approximately one-third of genes had differential expression). To validate the RNAseq results, we randomly assessed the differential expression of 10 genes using qRT-PCR, confirming the accuracy of the RNAseq data (Figure 7). Gene ontology (GO) enrichment analysis showed that differentially expressed genes were involved in a wide-variety of biological processes, cellular components, and molecular functions, including 21 distinct pathways (Figure 6). Therefore, we hypothesize that the RAYM_RS09735/RAYM_RS09740 TCS is a global expression regulator that controls the expression of virulence genes, thus affecting the pathogenicity of *R. anatipestifer*.

KEGG pathway analysis showed that RAYM_RS09740 is a PhoP protein, indicating that RAYM_RS09735 and RAYM_RS09740 form a PhoP/PhoR TCS. PhoP/PhoR TCSs have been implicated in several biological processes in Gram-positive bacteria, such as *B. subtilis* (Hulett et al., 1994), *Streptomyces lividans* (Sola-Landa et al., 2003), and *M. tuberculosis* (Perez et al., 2001). Specifically, PhoP/PhoR has been shown to control respiration(Birkey et al., 1998), cell wall metabolism (Minnig et al., 2005), culture metabolism (Thomas et al., 2012), and biofilm formation (Bluskadosh et al., 2013). Most importantly, in many pathogens PhoP/PhoR TCSs have also been shown to regulate pathogenesis (Perez et al., 2001; Ryndak et al., 2008). PhoP and PhoQ TCS components have been reported in many Gram-negative bacteria, including *E. coli* (Eguchi et al., 2007), *Edwardsvilla* (Lv et al., 2012), and *Salmonella* (Tran et al., 2016). PhoP/PhoR is also an important TCS in *M. tuberculosis* and is a key player in virulence (Ryndak et al., 2008). However, PhoP/PhoR TCSs have not been previously observed in Gram-negative bacteria. Our data shows that a putative PhoP/PhoR TCS is involved in *R. anatipestifer* virulence, although further phosphotransfer profiling will be required to validate RAYM_RS09735/RAYM_RS09740 as a PhoP/PhoR TCS.

Our data also suggest that RAYM_RS09740 can act as a transcription factor and plays a role in regulating genes expression, although there are likely other TCSs present in *R. anatipestifer*. This is based on our observation that, after the deletion of *RAYM_RS09735* and *RAYM_RS09740*, other TCSs regulated gene expression. We hypothesize that cross-talk and -regulation may occur between a multitude of two-component systems in *R. anatipestifer*, similarly to other organisms. For example, the transcription factor NemR is a redox-regulated transcriptional repressor in *E. coli* (Gray et al., 2013) and a carbohydrate-responsive regulatory protein (BadR) is a transcriptional repressor of *rpoS* in *Borrelia burgdorferi* (Miller et al., 2013).

The PhoP/PhoR TCS we identified was initially predicted to be part of the phosphate (Pi) stress-response in *R. anatipestifer*. PhoR acts to sense environmental phosphate (Pi) levels in bacteria through the ABC-type phosphate-specific transport (Pst) system and the protein PhoU. PstS is a periplasmic protein that binds Pi with high affinity and PhoP induces the promoter activity of *pstS*. We found with RNAseq and qRT-PCR that the mRNA expression of *pstS* was 8-fold higher (Figure 8A) in the mutant strain and different from other Gram-negative bacteria. Therefore, RAYM_RS09735 and RAYM_RS09740 may act more like the PhoP/PhoR system of *M. tuberculosis*. Additionally, *M. tuberculosis* PhoP/PhoR does not respond to phosphate starvation and is similar to the PhoP/PhoQ TCS in *Salmonella*. In *Salmonella* PhoP/PhoQ TCS, PhoQ senses low Mg^2+^ levels, and PhoP activates expression of genes encoding high-affinity Mg^2+^ transport systems (Walters, 2006). We aim to investigate the environmental factors sensed by *R. anatipestifer* PhoP/PhoR in future work.

Further, DEG analysis showed that the expression of several other signal transduction system genes was altered in the YMΔRS09735/RS09740 strain. It is likely that PhoP can influence the expression of transcription factors in other signal transduction system and there is cross-talk or cross regulation between PhoP/PhoR and other TCSs in *R. anatipestifer*. As more than one third of genes demonstrated differential expression, it is unlikely that the PhoP/PhoR TCS directly regulates all of these DEGs. However, the TCS we identified may affect wider gene expression by influencing the expression of other transcription factors in a variety of signal transduction systems.

Additionally, the CRISPR-Cas bacterial immune system has been shown to be present in *R. anatipestifer* (Chamnongpol et al., 2003; Zhu et al., 2016) and we found that the expression of *cas9, cas1*, and *cas2* were all different in the YMΔRS09735/RS09740 mutant, compared to the wild-type. Recent studies have reported that quorum sensing has a role in controlling the *Pseudomonas aeruginosa* CRISPR-Cas adaptive immune system (Høyland-Kroghsbo et al., 2016) and the TCS BfiSR affects the production of proteins involved in virulence, post-translational modification, and quorum sensing (Petrova and Sauer, 2010). We therefore reasoned that TCS might also affect the CRISPR-Cas immune system of *R. anatipestifer*.

We found 13 genes encoding hypothetical proteins that were downregulated more than 4-fold. At present, M949_RS01915, M949_1360, AS87_01735, AS87_03730, M949_1556, and AS87_04050 are all hypothetical proteins but have been reported to associate with virulence in *R. anatipestifer*. Bacterial lipoprotein (BLP) and the hydrolase Nlp/P60 were both found to be significant down-regulated in the mutant YMΔRS09735/RS09740 strain (Figure 8B). Nlp/P60 is involved in cell growth and division, autolysis, and invasion (Xu et al., 2015). Bacterial lipoproteins on the cell surface include an important class of virulence factor that is expressed by many pathogens. BLP can promote TLR2 expression, TLR2-induced NF-κB activation, and IL-6 production, leading to immune inflammation (Buddelmeijer, 2015). The clinical symptoms of *R. anatipestifersis* are characterized by fibrinous pericarditis, glissonitis, airbag inflammation, and meningitis. Our pathological results demonstrated that the damage induced by the mutant YMΔRS09735/RS09740 strain to the heart, brain, and spleen was significantly less severe than the wild-type strain (Figure 4). We therefore hypothesize that *R. anatipestifer* reduced expression of BLP and the hydrolase Nlp/P60, subsequently affecting inflammation in ducklings.

Other downregulated genes we identified included transcription factors, metabolic genes, multiple molecular chaperones, and TonB-dependent receptors. The primary role of DNA binding proteins and transcription factors is to regulate the expression of the genes and RAYM_RS09740 may regulate transcription by modifying the expression of these transcription factors. Molecular chaperone proteins assist in the folding of nascent polypeptide chains and prevent the aggregation of denatured proteins (Hartl and Hayerhartl, 2002). We found that transcript for the molecular chaperones *dnaJ, dnaK, grpE, clpP, tir*, and *skp* were all downregulated in the mutant YMΔRS09735/RS09740 strain, suggesting that the RAYM_RS09735/RAYM_RS09740 TCS may regulate the expression of the chaperone genes to influence the severity of *R. anatipestifersis*. The DnaK molecular chaperone system includes DnaK, DnaJ, and GrpE (Mayer et al., 2000; Ben-Zvi and Goloubinoff, 2001; Genevaux et al., 2007) and is related to stress tolerance and virulence in *Salmonella*. DnaK can also regulate the expression of virulence islands and a DnaK mutation strain was found to be largely avirulent (Takaya et al., 2004). ClpP was found to be a virulence-related factor in *Actinobacillus pleuropneumoniae*, with a *clpP* gene deletion strain demonstrating decreased biofilm production (Xie et al., 2013). We also found that TonB-dependent receptors were downregulated. These receptors have been reported to associate with hemin iron acquisition and *R. anatipestifer* virulence.

In this study, RAYM_RS09735 and RAYM_RS09740 were predicted to be components of a PhoP/PhoR TCS in *R. anatipestifer* using bioinformatics analysis. Furthermore, deletion of the *RAYM_RS09735* and *RAYM_RS09740* genes significantly decreased *R. anatipestifer* virulence. RNAseq analysis showed that RAYM_RS09740 is a global expression regulator in *R. anatipestifer*. We also demonstrated that the RAYM_RS09735/RAYM_RS09740 TCS contributes to virulence, signal translation, and the CRISPR-Cas adaptive immune system of *R. anatipestifer*. BLP and the hydrolase Nlp/P60 were predicted to be two virulence factors involved. Although common in Gram-positive bacteria, the predicted PhoP/PhoR TCS we have putatively characterized is the first such TCS identified in Gram-negative bacteria. Our study is also the first to report this TCS in *R. anatipestifer*, providing new insight into the role of TCSs in regulating *R. anatipestifer* virulence.

## Ethics statement

All the animal experiments were carried out in accordance with the recommendations in the Guide for the Care and Use of Laboratory Animals from Research Ethics Committee, Huazhong Agricultural University, Hubei, China. All procedures performed in studies involving animals were in accordance with the ethical standards of the institution or practice at which the studies were conducted.

## Author contributions

Funding acquisition: ZL. Investigation: YW. Methodology: YW, TL, and XY. Project administration: SL, ML, SH, and DB. Resources: ZZ. Supervision: ZL. Validation: ZL. Visualization: YW. Writing—original draft: YW. Writing—review's editing: ZL.

## Funding

This work was supported by grants from the Wuhan Science and Technology Bureau (No. 2015020101010070 to ZL) and Natural Science Foundation of Hubei Province (No. 2015CFB268 to ZL).

### Conflict of interest statement

The authors declare that the research was conducted in the absence of any commercial or financial relationships that could be construed as a potential conflict of interest.
